# The American Institute of Homœopathy

**Published:** 1884-06

**Authors:** F. R. McManus

**Affiliations:** Baltimore


### "THE AMERICAN INSTITUTE OF HOMŒOPATHY."

F. R. McManus, M. D., Baltimore.

In the March number of The Homœopathic Physician appeared an article, with the above title, from the pen of our justly distinguished colleague, P. P. Wells, M. D., of Brooklyn, New York, giving an account of the object for which the Institution was established, and was to be perpetuated, and stating, fearlessly, and truthfully, its short-comings. I agree with Dr. Wells in all that he has written upon the subject.

In the year 1837 I had the good fortune, after having been occupied eight and a half years in practicing medicine according to the theories and practices of the then dominant school, to have commenced an investigation of Homœopathy, which investigation I have continued, unremittedly, ever since. After having been thus engaged for seven years, I received an invitation to attend a meeting of homœopathic physicians, to be held in the city of New York, and to form what has been known as the "American Institute of Homœopathy." I attended, and am happy and proud to say that on the 10th day of April, 1844, I

was one of the twenty-three who commenced that successful undertaking. All are acquainted with that success—Homœopathy's enemies as well as friends. At its formation the material founders were unquestioned homœopathists—*Hahnemannians of the true grit.* If that glorious Institute has not fulfilled its destiny, and the expectations and requirements of many of its truest members and friends, it may be attributed, largely, to the great desire that has prevailed of rather increasing its size than to have had the more important object in view, the mental, literary, and medical capabilities of its applicants for membership. It may here be asked—"for what purpose the Board of Censors?"—I have attended every meeting of the Institute, far and near, since its formation, *but one*, and at every meeting have been honored with the responsible position of a member of the Board of Censors *but one*, and have always regretted the want of knowledge of the proficiency of applicants for membership by the Board of Censors. Under the mode of application for membership, every applicant is required to have his or her application guaranteed by three members of the Institute, for his medical diploma, name and location of the college of which he or she are graduates, and the year of such graduation, his moral and professional standing, etc. The Board of Censors are to be satisfied of the applicant's knowledge of Homœopathy, and then to recommend his or her election to membership. In regard to the first requirement, any physician (member) can obtain the indorsement of two others, who do so *as a compliment* and *know nothing of the status of the applicant!*

As regards the requirements of the Board of Censors as to the applicant's knowledge of Homœopathy, in nine cases out of ten the applicants do not appear before the Board of Censors, or before the Institute until elected, and no idea can be had of the applicant's eligibility in any respect. Again, of late years very many young physicians who may have had diplomas for about two or three months, who have really no knowledge of what system they will be the practitioners, and are applying for the testimonial of the great American Institute of Homœopathy to their moral and social and professional standing and as experts in Homœopathy, when they do not understand its first principles or the application of those principles to practice.

It can now be easily understood what an opportunity the American Institute of Homœopathy has offered to many to impose on the credulity of their several locations; to cover their ignorance or cupidity. They glory in the name of homœopathists, but are of that sensible class who, alone, are rationalists.

The Board of Censors require that every application for membership shall be in the handwriting of the applicant, and *that every member who indorses his eligibility shall have a personal knowledge of the applicant and the facts to which he attests.*

